# Performance and jump-to-jump development in the first female ski flying competition in history

**DOI:** 10.3389/fspor.2024.1366042

**Published:** 2024-05-01

**Authors:** Ola Elfmark, Øyvind Sandbakk, Magnus Brevig, Gertjan Ettema

**Affiliations:** ^^1^^ Department of Neuromedicine and Movement Science, Centre for Elite Sport Research, Norwegian University of Science and Technology, Trondheim, Norway; ^^2^^ Norwegian Ski Federation, Oslo, Norway

**Keywords:** female, ski jumping, ski flying, sex-difference, physical performance score

## Abstract

In 2023, for the first time in history, the international ski and snowboard federation (FIS) arranged an official ski flying competition where the 15 highest ranked women were allowed to participate. This study investigated jump-to-jump performance development in female ski flying, with men’s results used as reference data. Official FIS data from all six jumps of women were evaluated together with the eight jumps by men. Performance was evaluated by a score, where the distance points compensated by wind were divided by take-off speed, enabling performance to be evaluated across jumps and sexes. Women improved performance by 96% from the first to the sixth jump, with two major leaps; from the first to the second jump and from the first to the second day. In contrast, men mainly improved from training to competition. The best women had performance scores equivalent to the 10–20 best ranked men and the sex-difference between the top 3 athletes was 26.2%. This difference was thereafter compared to similar results in the normal and large hill World championship in Planica 2023, in which sex-differences between the top 3 were 8.6% and 14.6% in normal and large hill. This historical competition showed the importance of gaining practical experience with ski flying on performance, exemplified by the large improvement of female athletes. This, together with the enlarge sex-differences in large compared to normal hills, indicates that female ski jumpers have a particularly large improvement-potential in ski flying and must gain specific experience on this through traning and competitions.

## 1 Introduction

Ski jumping was one of the disciplines performed in the first Winter Olympics in Chamonix, 1924 (1). Although ski jumping has evolved tremendously over the years, the ability to jump as far as possible has always been imperative. The first recorded world record dates back to 1908 (9.4 m Olav Rye), while the first jump above 100 m was achieved by Sepp Bradl in 1936 and the first jump above 200 m by Tony Nieminen in 1994. Currently, Stefan Kraft holds the world record of 253.5 m set in 2017. Today, ski jumping competitions are separated into three categories based on the size of the hill, normal hill (NH) (hill size 85–109 m), large hill (LH) (hill size 110–184 m) and ski flying (SF) (hill size >185 m). The development of female ski jumping has been limited by restrictions in terms of allowance to compete in general, and the size of the hill in particular. Women competed in the World Cup (WC) for the first time in 2011 and had their first World Championship in LH in Obersdorf in 2021. Before 2023, SF events for women had not yet been performed.

The main argument for not allowing women to jump in large hill sizes have been a suspicion that they have a higher injury risk compared to their male counterparts. However, this is not based on evidence (2). Nevertheless, for the first time in ski jumping history, the international ski and snowboard federation (FIS) decided to hold an official SF competition for the 15 top ranked female ski jumpers during the Raw Air in Vikersund 2023. A total of 90 jumps were performed, where Yuki Ito became historical as the first female to jump in a FIS SF competition. Ema Klinec won the competition and set the female world record of 226 m. SF cannot be performed outside regulated competitions, enabling an interesting and rare scenario where the performance of experienced female ski jumpers could be observed from when they perform the sport for the first time. In addition, the comparison of performance and performance development of male counterparts, who mainly compete in LH and where most have experience from SF competitions, are also of interest.

So far, research on female ski jumping is limited to a few exceptions (3, 4). In contrast, ski jumping research on men dates back to 1926 (5) and has been a relatively popular scientific topic. The consensus in previous research is to split a ski jump into different phases (inrun, take-off, glide preparation, steady glide, landing preparation and landing phase) where the take-off phase is considered to be most important for performance (6, 7). However, larger hill sizes, higher competition speeds and longer aerial times have made manipulation of aerodynamic forces more important and extensively investigated in previous years (6–12). The aerodynamic forces are deemed to be particularly influential in SF, which is a competition form rarely mentioned in the scientific literature (13, 14).

Hill size influences the key features associated with ski jumping performance which may also affect male and female ski jumpers differently. Elfmark et al. (6) found that clear differences exist between the NH and LH regarding the influence of key variables in the aerial phase. In the NH, performance was found to be largely determined by the take-off and glide preparation, while the steady glide (especially having a high ratio between aerodynamic lift and aerodynamic drag) was found to be as important as the take-off in the LH. The factors of importance in a LH are assumed to be of even higher importance in a SF, where the aerial time is around 8 s compared to 4 s in a LH (6). Women mainly compete in NH (61–77% of competitions in the two previous seasons) while men almost exclusively (>90% of competitions) compete in LH and SF. Comparing performance of men and women in ski jumping are complex as women usually start from higher start gates to achieve a higher take-off speed, and the take-off speed and jump distance are closely related (11). Hence, to assess women’s performance, and compare with men, one needs to adjust distance for speed and wind conditions.

Given the limited data on SF in general and female ski jumping particularly, the aim of this study was to investigate the jump-to-jump performance development in female ski flying, using men’s data as reference. It was hypothesized that female performance would improve jump-to-jump as they gained experience in ski flying, and that the performance difference between women and men would gradually decrease.

## 2 Method

### 2.1 Data background

Official and open access FIS data from training and competitions were used to evaluate performance and the associated jump to jump development. From every official FIS round, data on jump length, start gate, take-off speed, average wind and total point score is provided. All data used for this investigation can be found at FIS homepage (15). During the three days of competition, men performed eight jumps, while women performed six jumps the last two days. To indicate different jumping rounds, an abbreviation is used where the sex together with the round is noted, for example M1 indicate men’s jumping round 1. For all female jumps n=15, n varied for the men depending on the type of jump (n=50 in M1, M2 and M6, n=40 in M3, M4 and M7 and n=30 in M5 and M8). An overview over the jumps, which day the jumps were performed, type of round, and wind condition are displayed in Supplementary Table S1. The sex-difference was compared to the NH and LH World Championship competition in Planica 2023, to further assess the sex-differences observed in SF. The average performance in NH and LH during the 2022/2023 WC of the 15 females will also be discussed to put the individual performance in context, all data used can be found at FIS homepage (16).

### 2.2 Physical performance score

In ski jumping, the main performance criterium is the jump distance. However, this distance depends not only on the athlete’s skill, but also on wind conditions and speed at take-off (7, 8). Take-off speed partly depends on the athlete’s skill, but also on the distance travelled along the inrun. This distance is determined by the choice of the start gate (position up the inrun), which is set by the jury but can be changed for tactical reasons by the coach or safety reasons by the jury during the competition. The wind compensation system is used to ensure fairness by adding or subtracting points for favorable or unfavorable wind conditions (17). The final performance based on skilled execution, is therefore expressed in a point-based manner where points are achieved from the jump distance ($P_{d}$), corrected for wind condition ($P_{w}$) and possible change in start gate. Subsequently, style points are added to this score based on the rationale that style reflects skilled performance (18). For example, style points are deducted for not landing with a “Telemark” technique (one foot ahead of the other, knees bend).

In the current study, we aimed to isolate the skilled performance regarding take-off action and flight, resulting in jump distance. Because take-off speed affects jumping distance and was not a performance factor of interest, we normalised the wind corrected points for distance ($P_{d}+P_{w}$) for take-off speed ($V_{t}$):

(1) $$S_{\;pp}=\frac{P_{d}+P_{w}}{V_{t}}.$$

Note that $P_{w}$ may be negative for favourable wind conditions. The parameters used in Equation 1 are provided on official result sheets from FIS for all formal competitions indefinitely and enables studying performance over a wide range of competitions, seasons and across sex (15).

## 3 Results

### 3.1 Performance development

**Figure 1:**
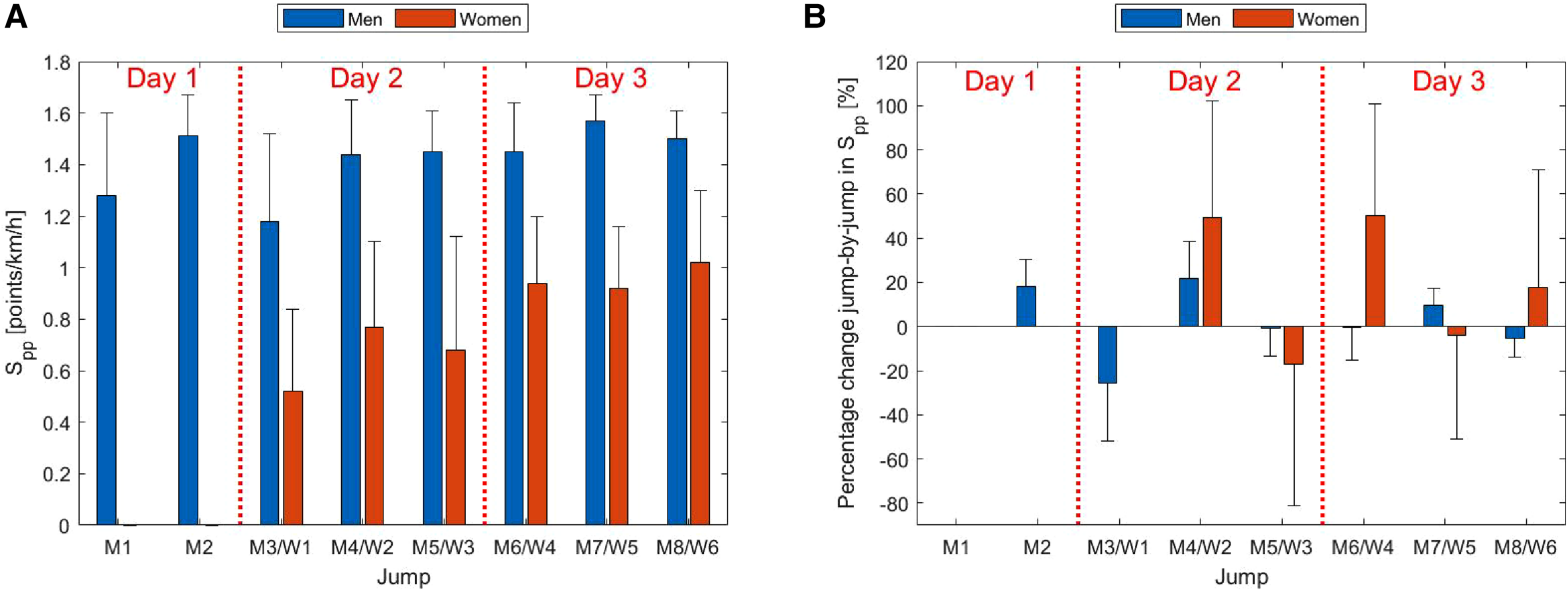
Physical performance scores ($S_{\;pp}$) and percentage improvement jump-by-jump (M1–M8 and W1–W6) for each sex during the ski flying event. The average $S_{\;pp}$ per jump is displayed in (**A**) and the percentage change from jump-to-jump in (**B**). For example, W2 display the change between W1 and W2, W3 the change between W2 and W3. Men are displayed in blue and women in orange.

In Figure 1, the average $S_{\;pp}$ of each jumping round and the relative percentage change from jump to jump (as indicator for development) for each sex are displayed.

Women experienced a 50% improvement from the initial to the second jump, with a slight reduction in improvement observed in the transition to the final jump of the first day. Furthermore, there was an additional performance boost between the days, as they demonstrated an average improvement of 86% on day 2 (W4–6) compared to the first jump (W1). Consequently, female ski jumpers exhibited two performance leaps, the first occurring between the initial and second jumps, and the second between the first and second day of jumping. The average improvement from the first to the last jump amounted to 96%. However, the high standard deviation in the female ski jumping group indicates a large internal variation. Notably, the improvement for women was 4–5 times greater than that for men. Men generally displayed a lower $S_{\;pp}$ in the two training rounds (M1 and M3), rounds that did not contribute to the competition. Men exhibited an average improvement of 16% in rounds 4–8, all of which were included in the overall competition.

**Figure 2:**
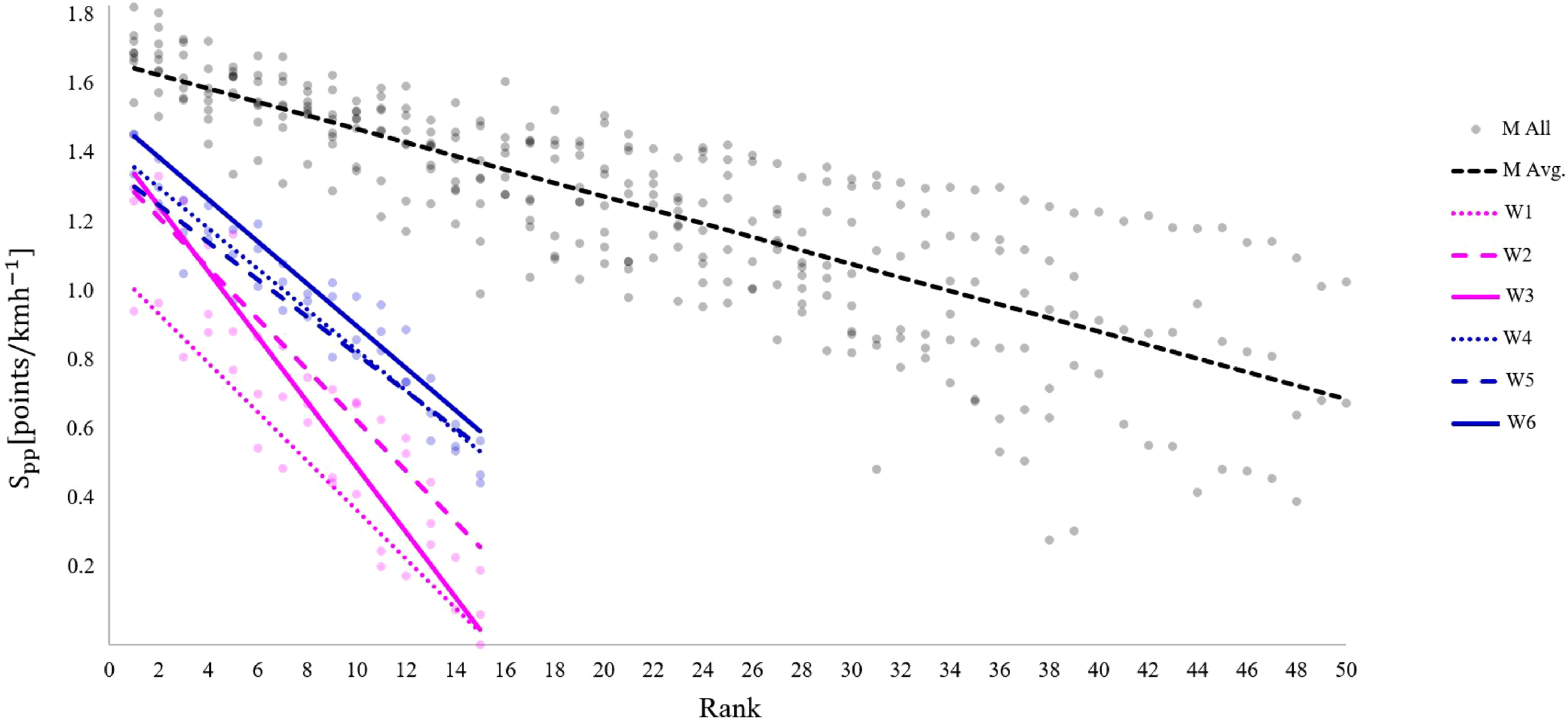
Relationship between physical performance score ($S_{\;pp}$) and rank per jump. W1–W6 indicate the six rounds of female jumps during the Raw air ski flying tournament and are displayed together with all male jumps (black) as a reference. The regression lines for each jump are grouped in the colors magenta and blue for each day.

The $S_{\;pp}$ from all female jumps vs. the competition ranking, and the regression of each round together with all male jumps are displayed in Figure 2.

Adusjed *R*^2^ values were 0.93, 0.96, 0.87, 0.93, 0.90 and 0.92 for W1, W2, W3, W4, W5 and W6, respectively. The difference between the athletes decreased from the first to the second day. The constant of the linear regression increased through the first three jumps and the highest overall performance was found in the last jump (W6), where the best women performed similar to the 10–20 best ranked men.

### 3.2 Performance differences across hill sizes

The average SF $S_{\;pp}$ of top 3, 1–15 and 13–15 were compared with data from the World Championship in NH and LH Planica 2023 in Table 1. Here, men and women are compared in the same hills.

**Table 1:** Average physical performance scores ($S_{\;pp}$) and standard deviations among the top 15 ranked ski jumpers, of the 3 best (ranked 1–3) and 3 worst (ranked 13–15) of the World Championship competitions in the normal and large hill in Planica 2023 and the ski flying competition on day 3 of the Raw air tournament in Vikersund 2023.

Athletes	Normal hill	Large hill	Ski flying
	[pt/km h^-1^]	[pt/km h^-1^]	[pt/km h^-1^]
Women ranked 1–3	0.81±0.04	0.82±0.08	1.30±0.11
Men ranked 1–3	0.88±0.04	0.94±0.04	1.64±0.08
Women ranked 1–15	0.74±0.05	0.71±0.08	0.97±0.26
Men ranked 1–15	0.84±0.06	0.88±0.05	1.53±0.11
Women ranked 13–15	0.68±0.03	0.64±0.05	0.66±0.14
Men ranked 13–15	0.80±0.09	0.82±0.03	1.48±0.07

The performance difference between the top 15 men and women was smallest for the NH (13.5%) and the difference increased to 23.9% and 57.7% for the LH and SF, respectively. Similar trends were observed when comparing top 3, but sex-differences were roughly halved, being 8.6%, 14.6% and 26.2% for the NH, LH and SF respectively. The difference between men ranked 1–3 and 13–15 were quite similar at 10.0%, 14.6% and 10.8% for the three hill sizes. This difference increased for women, being 19.1%, 28.1% and 97.0% for the NH, LH and SF, respectively.

## 4 Discussion

The primary purpose of this study was to investigate jump-to-jump performance development during the first official SF event for women, where men’s results were used as reference data. The main findings were that women improved their performance 4–5 times more than men and had an average 96% improvement from the first to the sixth jump. Women had two performance leaps: one between the first and second jump and the other between the first and second day. The best female jumpers had scores equivalent to 10–20 best ranked males after six jumps. At the same time, the sex-difference in performance increases with hill size, from 8.6% in NH to 26.2% in SF among top 3 jumpers. Indicating that there still is a way to go for female ski jumping, especially when jumping in the larges hill sizes.

### 4.1 Performance development

Due to FIS safety regulations, nobody is allowed to perform SF outside of an official arranged FIS competition, and women have not been allowed to compete in SF. This gave an interesting scenario where none of the 15 ski jumpers had tried SF before the first jump in this collection (W1). Hence, this study has been able to quantify and describe the performance of the female ski jumpers and the development through their first six jumps, using the $S_{\;pp}$. These data enable us to understand how fast they learned or adapted to SF and how they progressed jump-by-jump.

In this case, women improved their $S_{\;pp}$ by 50% from the first to the second jump (W1 to W2), with a similar performance improvement from the last jump of the first day to the first jump of the second day (W3 to W4), 86% on average from W1 to day 2. Thus, after only one jump, women improved their performance significantly and continued this improvement into the next day. Video analysis is used by most coaches in ski jumping to capture the take-off (19). Between the days, ski jumpers have time to analyse video together with their coaches, which most likely has been influential for their development. Different techniques can be used to achieve a good results in ski jumping (18), however a slight change in technique may have been nessesary for the women without experience. Such changes are easier to adapt after video analysis and the use of imitation jumps that are commonly used for technique training in ski jumping (20). Altogether, women had an improvement of 96% from the first to the sixth jump. On the second day, the largest improvements were found in the bottom half of the group, i.e., the difference between the jumpers decreased.

A similar performance trajectory was not observed for the men, where nearly all had performed SF before. The male ski jumpers performed in general 16% better in qualification and competition jumps, which all counted in the overall Raw air tournament, compared to the training jumps (M1 and M3). Only small differences were observed between the qualification and competition jumps. Ski jumping is a sport where the equipment is important for the overall performance, and one reason for the difference in performance may be that the ski jumpers did not used equipment (suits and skis) of competition quality in training, which is known to be influential (1).

### 4.2 Performance differences across hill sizes

The performance difference between the female ski jumpers ranked 1–3 and 13–15 was largest for SF and decreased with hill size, dissimilar to men who had similar internal differences. Female ski jumping is a relative new sport. Hence, there are fewer female than male ski jumpers and the sport is developing more for women than men. Women had never tried SF before this competition, whereas men have been competing in SF for several years. Men have their majority of competitions in LH and SF which may make men more homogeneous as a group compared to women, i.e., the internal difference is more stable across the hill sizes. Although an increasing number of competitions are performed in LH for women, during a WC season, women alternate between the hill sizes more than men. As explained by Elfmark et al. (6), there are clear differences in how key variables through the take-off and aerial phase influence performance in these two hill sizes. Thus, some women may specialise for jumping in NH whilst others in LH, which could explain a larger performance difference in competitions.

Women who performed well in the SF event tended to be the same athletes that in generally performed better in LH than in NH in WC competitions. As an example, ranking 1, 2 and 3 in the current SF event averaged 4.8, 9.1 and 5.8 better places in LH competitions compared to the NH competitions during the WC season 2022/2023, respectively. One of the most influential factors in LH and SF is to increase the horizontal speed (6). A jumping technique suited for NH jumping will aim to reduce vertical speed through the aerial phase, which also causes a decrease in horizontal speed (6). Hence, based on their earlier WC results, some of the female ski jumpers seemed to over perform whilst others under performed in SF. However, this may be explained by their technique being hill specific, i.e., focusing either on increasing horizontal speed (advantage in SF) og reducing vertical speed (disadvantage in SF).

As women had two performance leaps during the days, the average of the competition jumps (W5 and W6) was used to compare their performance with the average male performance. The $S_{\;pp}$ of the three best women were equivalent to men ranged from 10–20, whilst the women ranked 13–15 had scores equivalent to being 40–50 ranked by the males. It should be noted that this comparison is merely based take-off speed not which gate that speed is achieved from. Thus, the best women would not have been expected to be in the 10–20 range overall in a male competition, as they would have produced less take-off speed if starting from the same gate. Furthermore, they traditionally would have gained fewer style points. Thus, in our opinion, a virtual comparison of true competition results is inflated.

In this context, one argument against women’s SF completion have been that women need dangerously high take-off speed to obtain adequate jump lengths. Based on this analysis, they may not need much higher take-off speed, only higher starting gate. Taking 220 m as reference value, five out of 90 jumps (5.5%) by women were over this distance with an average take-off speed of 101.2 ± 1.6km h^-1^. Men had 79 of 322 jumps (24.5%) over 220 m with an average take-off speed of 98.6 ± 1.5km h^-1^. Thus, from a practical perspective, women may not need much higher take-off speeds, 2.6km h^-1^ in this case, than men to obtain jump lengths that are well respected and thereby attractive sports-wise. Moreover, all 90 female SF jumps from this historical event, within the scope of the two days, were performed without any crashes or injuries. This stands in contrast to the male ski jumpers in the same event, where several crashes were reported both by competing and trial jumpers. Safety concerns in ski flying should always be of the highest importance, as it is a dangerous sport. However, the special safety concern regarding women, which have led to restrictions, should be evaluated as the physical performance of these first jumps do not indicate any additional safety concern based on sex.

### 4.3 Methodological considerations

The $S_{\;pp}$ used in this study was based on official FIS-data of jump distance, wind compensation and take-off speed. This enabled us to evaluate performance from jump-to-jump and to compare sexes. This score has not been used in research previously, and it is worth emphasizing that the score does not give an exact measure of overall competition performance, but a measure of the physical performance of the ski jump. Hence, how many distance point, compensated by wind, a ski jumper can produce per take-off speed. The performance comparison with the male counterparts for example is only used to get a perspective on the female performance levels, not to consider if they would have been able to compete in a male competition, which the $S_{\;pp}$ enable us to do.

The wind compensation is included in the $S_{\;pp}$ to compensate a performance for wind conditions. The validity of the wind compensation algorithm used by FIS, i.e., its fairness, is a matter of debate. In the current investigation, the wind condition was fairly similar between the sexes and any correction model would have had little impact on the companions. Still, on average, men jumped in slightly more difficult conditions (0.19 ms^-1^ compared to 0.31 ms^-1^ for females). The algorithm is developed based on knowledge on aerodynamics (17), and there is currently no better alternative for this estimate. Hence, the comparisons of $S_{\;pp}$ across jumps and sex are improved with the wind compensation included in the score compared to not taking wind into account.

$S_{\;pp}$ explains how many distance point, compensated by wind, an athlete is able to achieve per take-off speed. Together with the $S_{\;pp}$, an overall performance can be explained by two factors; obtaining as high take-off speed as possible from the gate set by the jury and achieve as many style points as possible. These two do also need to be evaluated for a complete performance analysis. Nevertheless, a performance score such as $S_{\;pp}$ enables us to analyse ski jumping performance to a new extent, but an in-depth validation should be performed in future research.

## 5 Summary

In the present study, where novel data from the first female SF competition are presented, we used a physical performance score ($S_{\;pp}$) to evaluate performance and jump-to-jump development of female ski jumpers with the corresponding results of men as a reference. Women improved their performance 4–5 times more than men and had an average 96% improvement from the first to the sixth jump, where the male ski jumpers merely improved between training and competition. The female ski jumpers had two performance leaps; from the first to the second jump and between the two days of jumping. The SF performance sex-difference between top 3 for the two last female jumps was 26.2%, compared to 8.6% and 14.6% in the NH and LH. Altogether, women were more than capable to compete in SF and their SF performance increased with experience, and thereby reduced the sex-difference. The large improvement in jump-to-jump development in women, combined with the increase in sex differences with hill size, demonstrates that female ski jumpers who wish to maximize their potential on LH and in SF must gain specific experience through training and competitions.

## Data availability statement

Publicly available datasets were analyzed in this study. This data can be found here: https://www.fis-ski.com/DB/general/event-details.html?sectorcode=JP&eventid=51540&seasoncode=2023.

## Ethics statement

Ethical approval was not required for the studies involving humans because the data used from this study was official and open FIS data from competition found online. The studies were conducted in accordance with the local legislation and institutional requirements. Written informed consent for participation was not required from the participants or the participants’ legal guardians/next of kin in accordance with the national legislation and institutional requirements because Consent was not needed as the data used is open access online proided by FIS.
