# Supplementary material for: Performance and jump-to-jump development in the first female ski flying competition in history
Source: Front Sports Act Living. 2024 May 1;6:1366042. doi: 10.3389/fspor.2024.1366042 (PMC11094544; doi:10.3389/fspor.2024.1366042)
Supplement: Supplementary file 2 [file Datasheet1.pdf]

Supplementary Material

1 SUPPLEMENTARY DATA

Table S1 display an overview over the jumps in this investigation with explanation of which day the jumps were performed, type of jump, and wind condition. A supplementary excel sheet with data on all jumps (speed, distance points, start gate, wind compensation and S_{pp}) are also submitted together with this article. The abbreviation of the sex together with the round forms the abbreviation used for the jumps, for example: M1 = Male jump 1.

Table S1. Overview over the order and day of the jumps, type of jump, and average wind condition of the round for the male and female in the ski flying event.

Sex	Jump	Day	Competition type	Avg. wind $[\mathrm{m\,s^{-1}}]$
W	1	2	Training 1	0.42
W	2	2	Training 2	0.00
W	3	2	Training 3	0.96
W	4	3	Training 4	0.02
W	5	3	Competition, round 1	0.32
W	6	3	Competition, round 2	0.11
M	1	1	Training 1	0.90
M	2	1	Qualification 1	-0.02
M	3	2	Training 2	1.25
M	4	2	Competition 1, round 1	0.28
M	5	2	Competition 1, round 2	-0.36
M	6	3	Qualification 2	-0.19
M	7	3	Competition 2, round 1	-0.26
M	8	3	Competition 2, round 2	-0.09
